# Early detection of tuberculosis: a systematic review

**DOI:** 10.1186/s41479-024-00133-z

**Published:** 2024-07-05

**Authors:** Josef Yayan, Karl-Josef Franke, Melanie Berger, Wolfram Windisch, Kurt Rasche

**Affiliations:** 1 Department of Internal Medicine, Division of Pulmonary, Allergy and Sleep Medicine, Witten/Herdecke University, HELIOS Clinic Wuppertal, Heusnerstr. 40, 42283 Wuppertal, Germany; 2 Department of Internal Medicine, Pulmonary Division, Internal Intensive Care Medicine, Infectiology, and Sleep Medicine, Märkische Clinics Health Holding Ltd, Clinic Lüdenscheid, Witten/Herdecke University, Lüdenscheid, Germany; 3 Department of Pneumology, Cologne Merheim Hospital, Witten/Herdecke University, Cologne, Germany

**Keywords:** Early diagnosis, Screening, Diagnostic tests, Tuberculosis

## Abstract

Tuberculosis remains a significant global health challenge. Tuberculosis affects millions of individuals worldwide. Early detection of tuberculosis plays a relevant role in the management of treatment of tuberculosis. This systematic review will analyze the findings of several published studies on the topic of the early detection of tuberculosis. This systematic review highlights their methodologies and limitations as well as their contributions to our understanding of this pressing issue. Early detection of tuberculosis can be achieved through tuberculosis screening for contacts. Comprehensive health education for household contacts can be used as early detection. The in-house deep learning models can be used in the X-ray used for automatic detection of tuberculosis. Interferon gamma release assay, routine passive and active case detection, portable X-ray and nucleic acid amplification testing, and highly sensitive enzyme-linked immunosorbent assay tests play critical roles in improving tuberculosis detection.

## Introduction

Tuberculosis (TB) continues to be a health problem for many people. TB affects millions of people every year [1]. Despite significant advances in TB diagnostics and treatment, there remains a critical gap in our approach: early detection of TB. Early detection is greatly important in controlling the spread of TB [2]. In the ongoing battle against TB, the critical importance of early detection cannot be overstated. This systematic review introduces a meticulously refined concept of ‘early detection,’ explicitly encompassing the identification of latent TB infection and the diagnosis of TB in its initial symptomatic phase. Early detection refers to pinpointing individuals who harbor *Mycobacterium tuberculosis* without displaying active disease symptoms, identifying those at a heightened risk of progressing from a non-contagious, latent state to active disease. Concurrently, it involves recognizing the disease at the onset of its initial, often mild and nonspecific symptoms, such as persistent cough, fever, night sweats, or weight loss, to diagnose TB promptly before significant symptomatology and increased risk of transmission occur. By clarifying ‘early detection’ in this manner, we underline its indispensable role across the TB disease spectrum, emphasizing the need for timely and targeted diagnostic interventions. This approach sets a clear and actionable framework for healthcare professionals and public health initiatives, aligning with the imperative to prevent severe disease outcomes and curb the spread of TB. This is different from the general diagnosis of TB, which can be made at any stage during the progression of the TB. This systematic review explores the importance of early-detection methods for TB. Timely identification of TB cases is crucial for providing appropriate treatment, preventing transmission, and early treatment [3]. Early detection allows for prompt interventions and reduces the risk of severe complications [4]. It also curbs the transmission chain by enabling the identification and isolation of infected individuals, thus preventing further spread within communities [3]. Firstly, the major knowledge gap lies in the need for more effective strategies and tools for the early detection of TB. Traditionally, TB diagnosis has relied on sputum swab microscopy [5]. This investigation is widely available and cost-effective. However, it has limitations with regard to sensitivity [5]. Recent advancements in TB diagnostics have brought forth innovative tools that have revolutionized early detection [6]. These include molecular tests such as nucleic acid amplification assays (e.g., the GeneXpert MTB/RIF assay), which can rapidly detect the presence of TB bacteria and simultaneously determine drug resistance [7]. These molecular tests have demonstrated higher sensitivity and specificity, enabling earlier detection even in cases with low bacterial burden [8]. Secondly, addressing this gap is vital for several reasons. The new development of point-of-care diagnostics should help for early detection of TB [9]. These technologies have the potential to expand access to TB diagnostics in resource-limited settings [10]. Nevertheless, despite these advancements, several challenges persist with respect to the early detection of TB [11]. Limited access to diagnostics in developing countries hampers early detection of TB [12]. Delays in diagnosing TB can be attributed to various factors beyond stigma, lack of awareness, and reluctance to seek medical care [13]. These include limited access to diagnostic facilities, socio-economic barriers, and the complexity of the disease’s presentation, which can often mimic other less serious illnesses [13]. Timely TB diagnosis leads to more efficient patient care. It reduces the burden of advanced TB cases on health systems, and facilitates targeted public health interventions to control the spread of the disease [14]. Early detection of TB is crucial for effective disease management and control [1]. The sensitivity and specificity of TB testing has been improved by advances in diagnostic technology [15]. However, it remains imperative to address the challenges related to access, awareness, and stigma to achieve widespread and timely detection of TB cases [16].

The landscape of TB research is vast, with each domain – from molecular diagnostics to social aspects of contact tracing and active case finding – being extensively explored in various independent reviews. However, there exists a gap in the literature: a comprehensive synthesis that connects these discrete elements into a unified understanding of TB early detection. Our systematic review is poised to fill this gap, asking the question: “In what ways do the latest advancements across diagnostics, contact tracing, and active case finding synergize to enhance early TB detection, and what implications do they hold for global TB control?” Further research is necessary to find more accurate, and affordable diagnostic tools for early detection of TB [17].

## Materials and methods

### Literature search

A comprehensive literature search was conducted to identify relevant studies on the early detection of TB. Electronic databases, including PubMed, Embase, and Google Scholar, were searched using a combination of keywords and Medical Subject Headings terms (“early detection of tuberculosis”). Additional search terms were considered to ensure comprehensive coverage of the literature. Alongside “early detection of tuberculosis,” we included terms like “rapid TB diagnostics,” “novel TB detection methods,” and “emerging TB diagnostic technologies.” This expanded search strategy was employed to capture a broader range of relevant articles, including those not specifically using the term “early detection”. The search was limited to articles published in English up to June 2023.

### Study selection

Two independent reviewers screened the titles and abstracts of the retrieved articles to identify potentially eligible studies. Full-text articles were considered for further assessment if they were deemed to be potentially relevant or if there was uncertainty based on the abstract alone. Any disagreements between the reviewers were resolved through discussion or consultation with a third reviewer.

### Inclusion and exclusion criteria

Our objective was to emphasize a comprehensive approach to selecting pertinent literature on the early detection of TB that involves human subjects and presents primary data in peer-reviewed, English-language articles. The inclusion criteria were specifically designed to prioritize studies focusing on (1) early detection methods for TB, (2) research involving human subjects, (3) primary research data, (4) peer-reviewed status, and (5) publications in English. This framework aimed to identify literature with the highest relevance and potential to significantly impact the field. The selection was inherently guided by the articles’ innovation, relevance to current challenges in TB detection, and their potential to influence future research and public health strategies. This integrated approach ensured that our literature selection was both deliberate and impactful, focusing on distinguishing between general TB diagnosis and the specialized area of early detection strategies. These strategies account for the distinct methodologies and technologies involved in early TB detection. Our evaluation process involved a tiered review, starting with an initial screening based on title and abstract to ensure compliance with our primary criteria, followed by an in-depth assessment of the full texts to evaluate the articles’ innovation, relevance, and potential impact. This thorough review, conducted by a team of reviewers, aimed to minimize bias and fully appreciate each study’s contribution to advancing early TB detection. We focused on the novelty of the detection methods, their application to overcoming current public health challenges in TB management, and their capacity to shape future research and strategies. At the same time, studies primarily focused on diagnosing active TB without an explicit emphasis on early detection, as well as animal studies, case reports, editorials, and conference abstracts, were excluded to maintain a concentrated focus on early detection techniques. However, it is important to acknowledge that the distinction between diagnosing active TB and its early detection is nuanced. Many of the included studies indeed discuss diagnosing active TB within the context of early detection efforts, highlighting the essential link between these elements in effective TB management.

### Data extraction

The data collected for this systematic review were author, study publication year, study title, study location, study design, study population, sample size, type of tuberculosis diagnostic method evaluated, and key findings related to TB early detection.

### Data synthesis and analysis

The extracted data were analyzed to provide a systematic review of the different methods and approaches used for the early detection of TB. The results of the included studies were summarized based on the diagnostic methods, molecular testing, serological testing and symptom-based screening. Where applicable, summary measures such as sensitivity, specificity, and diagnostic accuracy were reported.

## Results

**Fig. 1:**
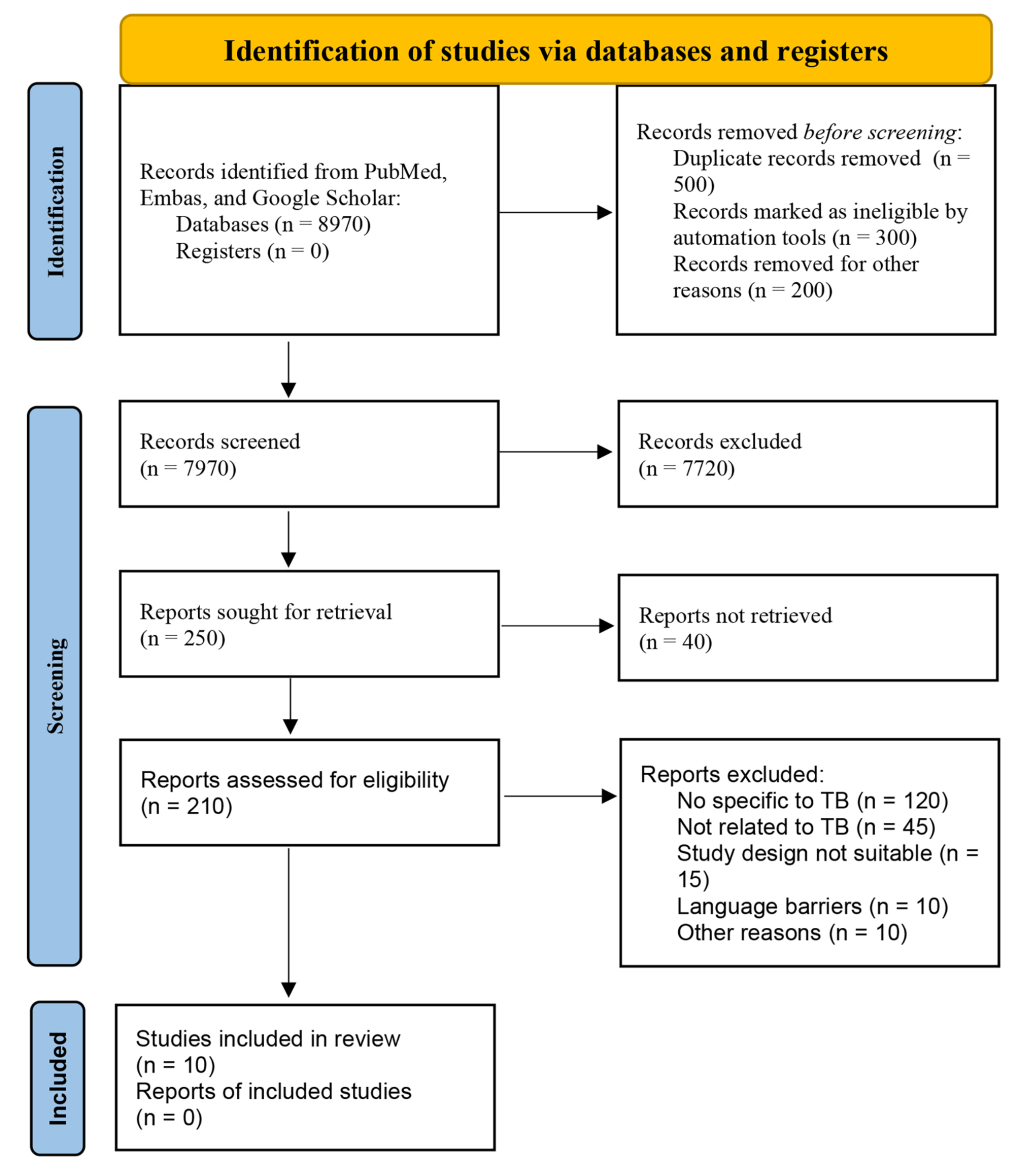
PRISMA 2020 flow diagram for the systematic review on early detection of tuberculosis, illustrating the stages from initial article identification to final inclusion in the systematic review

In our comprehensive search for medical literature on the early detection of TB, we encountered a significant volume of research, underscoring the field’s active investigation (Fig. 1). From the extensive array of articles reviewed across relevant medical databases, we selected 10 studies for citation in this study, focusing on their methodological contributions to early TB detection (Table 1). This focused selection was not indicative of a scarcity but a deliberate choice to deeply analyze studies that embody the forefront of innovation and impact within this area of research. Our objective was to delve into works that not only present new diagnostic methodologies but also have the potential to substantially influence future research directions and public health strategies. The selection and exclusion of articles were guided by a set of rigorous criteria, developed to ensure the inclusion of research that introduces novel diagnostic technologies, addresses current challenges in TB detection, and holds significant potential for impacting the field. Preference was given to studies showcasing innovation in early detection methods, those offering solutions to known gaps such as accessibility and applicability in low-resource settings, and research anticipated to shape future public health policies.

Conversely, our review process necessitated the exclusion of articles that did not meet these precise criteria. Studies focusing on the diagnosis of active TB at later stages, lacking empirical validation through peer review, published in languages other than English, or centered on narrower scientific inquiries such as animal studies, case reports, editorials, and conference abstracts were systematically omitted. This exclusion was pivotal in maintaining the integrity and focus of our systematic review on early detection methodologies.

The rationale behind this selective approach was twofold: to provide an in-depth analysis of key studies that represent significant advancements in early TB detection and to construct a narrative enriched by a broader examination of the field. While the primary analysis concentrated on 10 articles, our review’s scope was broadened through referencing a larger set of studies, enabling us to paint a comprehensive picture of the landscape of TB detection research. These additional references, although not subjected to detailed analysis, played a crucial role in situating our selected articles within the wider context of ongoing research efforts, thereby offering a nuanced understanding of the evolution and potential of early detection strategies in combating TB.

**Table 1:** Study characteristics, author, year of publication, study design, study population, sample size, type of TB diagnostic method evaluated, and key findings related to the early detection of TB. The Table 1 was designed to include only a select number of articles that meet specific criteria for systematic review, such as those that provide the most convincing evidence or focus on specific aspects of early detection of TB. The articles summarized in the text cover a broader range of topics in TB screening, while the table only includes studies that relate to a specific methodology or outcome

[Reference Number]	Author	Year of Publication	Study location	Study Design	Study Population	Sample Size	TB Diagnostic Method Evaluated	Key Findings for the Early Detection of TB
[18]	Gran et al.	2013	Norway	A cross-sectional study	Healthcare workers at an increased risk of TB infection	387	Interferon-gamma release assays, tuberculin skin test	Screenings for TB
[19]	Putra et al.	2019	Indonesia	Descriptive exploratory study	Household contacts with TB	498	Sputum examination (microscopic, Gene X-pert, culture), bacteriological confirmation and signs, screening symptoms, and chest X-ray examination	Screening TB contacts
[20]	Simi Margarat et al.	2022	India]	Hybrid model	Chest X-rays Images	662	Chest X-rays Deep learning model	Chest X-ray for screen-active TB with enhanced deep-learning model for automatic TB detection
[21]	Nijiati et al.	2022	China	A population-based retrospective study	X-ray images and corresponding clinical information were collected from individuals with and without TB.	9,268	X-ray analysis Diagnoses by experienced physicians based on the symptoms and the results of multiple tests and radiological examinations, sputum culture or smear tests, Xpert tests, chest X-ray films, interferon-gamma release assays, tuberculin skin tests	Deep Convolutional Neural Network (DCNN)-based artificial intelligence algorithm aimed at diagnosing TB through chest X-ray
[22]	Taki-Eddin et al.	2012	Syria	Prospective study	Suspected active pulmonary TB patients, sputum, three bronchial wash, three pleural fluid, peripheral blood	91	Interferon-gamma release assay, Ziehl-Neelsen smear, Lowenstein-Jensen’s egg-based culture, and real-time polymerase chain reaction	Interferon-gamma release assay was found to be more sensitive than the other conventional, molecular methods
[23]	Artawan et al.	2023	Indonesia	Randomized controlled trial	TB household contacts	428		Comprehensive health education to the improve the knowledge of household contacts
[24]	Abayneh et al.	2022	Southwest Ethiopia	A cross-sectional descriptive study	TB suspected patients	422	Gene Xpert-MTB/RIF assay	Gene Xpert assay
[25]	Eang et al.	2012	Cambodia	Community-based case control study	TB suspects	33,631	Chest radiography, sputum-smear microscopy	Routine passive and active case-finding using mobile X-ray machines
[26]	Wang et al.	2020	Taiwan	A prospective study	TB suspects	1,102	Sputum for ultrasensitive enzyme-linked immunosorbent assay	Nucleic acid amplification tests
[27]	Madukaji et al.	2021	Nigeria	A prospective study	Collected samples	150	Microscopy, phenotypic (Lowenstein-Jensen and liquid culture/DST), genotypic culture (Genexpert, LPA, fluorotype, Genetype	Early detection of pre-XDR TB with line probe assay in a country with high TB burden

**Abbreviations**: TB: Tuberculosis

### Screening for TB infection

Screening for TB is a valuable part of official public health strategies to monitor the spread of TB [18]. In reviewing the screening methods, several approaches can be considered [28].

The tuberculin skin test (TST) is commonly used for screening for early detection of TB [29]. The TST is also known as the Mantoux test [29]. It involves injecting a small amount of purified protein derivative (PPD) into the skin and then evaluating the size of the resulting induration after a specified time [29]. However, the TST has limitations. However, a false positive TST for TB can caused because a past vaccination with Bacillus Calmette-Guerin (BCG) [30]. Or previous infection with non-tuberculous mycobacteria can lead to false positive TST values [30].

The interferon-gamma release assay (IGRA) is another screening option. The IGRA measures the release of interferon-gamma by T cells in response to specific TB antigens [31]. IGRA tests, such as the QuantiFERON-TB Gold or T-SPOT [32], offer improved specificity compared to the TST and are not affected by BCG vaccination [33]. Although, they are more expensive and require laboratory facilities [34].

Chest X-rays can be used for TB screening, particularly for individuals with symptoms that are suggestive of pulmonary TB [35]. X-rays can discover nodules, infiltrates, or cavities [36]. However, X-rays have limited sensitivity, they are not suitable as screening for TB [36].

In recent years, molecular-based tests, such as the Xpert MTB/RIF assay, have gained prominence [37]. Such tests detect the genetic material of *Mycobacterium tuberculosis* [37]. Molecular tests offer rapid results with high sensitivity, making them valuable for screening of TB, especially in high-burden settings [37].

The choice of screening method depends on various factors. Screening for TB should be based on the population being screened, the resources available, and the purpose of the screening, such as active case finding or contact tracing [38]. A combination of different screening strategies, tailored to local circumstances, can enhance the effectiveness of TB control efforts [38].

### Screening TB contacts

The screening of individuals who have been in contact with TB patients plays a crucial role in early detection and prevention [19]. In this systematic review, we present the summarized findings from the previously published studies on screening TB contacts available on PubMed [39–41]. The reviewed studies consistently highlighted the importance of screening TB contacts for early case detection and prevention [39–41]. The most important insights from these studies included are as follows:

1. **Yield of active TB cases:** The screening of TB contacts led to the identification of a significant number of TB cases [39]. These individuals showed symptoms that were indicative of TB, such as persistent cough, weight loss, and fever [39]. The prompt identification and subsequent treatment of these cases contribute to reducing transmission and improving individual health outcomes [39].

2) **Detection of latent TB infection:** Screening also revealed a substantial proportion of TB contacts with latent TB infection [40]. These individuals showed no symptoms but tested positive for TB infection [40]. Identifying and treating latent TB infection cases are essential to prevent the progression to active TB disease in the future [40].

3) **Challenges with contact tracing:** The studies highlighted the challenges associated with conducting comprehensive contact tracing, including difficulties with locating and reaching all contacts, incomplete information with regard to contact details, and suboptimal follow-up rates [41]. These challenges emphasized the need for improved strategies and resources to enhance the effectiveness of contact tracing [41].

4) **Importance of TB Education:** The studies emphasized the significance of providing TB education to contacts, which includes information about symptoms, transmission, and preventive measures [42]. Education campaigns and counseling sessions were found to improve awareness of and adherence to screening and treatment protocols [42]. In sum, the results from the published studies reviewed through PubMed underscore the value of screening TB contacts for the early detection of active TB cases and the identification of latent TB infections. Contact-tracing efforts should be further strengthened, and educational interventions should be integrated into screening programs to maximize their impact with respect to TB control and prevention.

### Comprehensive health education to improve household contacts

A meta-analysis revealed that comprehensive health education interventions significantly improved the knowledge about TB among household contacts [43]. Furthermore, these interventions led to increased rates of TB screening and treatment completion among the contacts [43].

One study demonstrated that the community-based comprehensive health education program positively influenced household contacts’ understanding of TB symptoms, transmission, and preventive measures [23]. This led to an increase in early detection, the timely start of treatment and improved adherence to therapy among the contact persons [23].

The evaluation of the comprehensive health education intervention revealed significant improvements in the knowledge and awareness of TB among household contacts [42]. The intervention also resulted in higher rates of prompt healthcare-seeking behavior, increased uptake of preventive therapy, and reduced TB transmission within households [42].

The study demonstrated that comprehensive health education had a positive impact on the quality of life of TB-affected household contacts [44]. The intervention improved the quality of life, and the psychological well-being [44].

### Chest x-ray for screen-active TB with enhanced deep-learning model for automatic TB detection

According to the previous studies found in the medical scientific databases, there have been significant advancements in the use of enhanced deep-learning models for automatic TB detection in chest X-rays [21, 45, 46]. These studies have demonstrated promising results with regard to screening for active TB using computer-aided diagnosis systems. The application of deep-learning techniques has shown improved accuracy and efficiency in detecting nodules, infiltrates, and cavities, in chest X-ray images [47]. These findings suggest the potential of deep-learning models as a valuable tool for TB screening [48]. Multiple studies have demonstrated that deep-learning models exhibit high sensitivity and specificity to detecting TB in chest X-ray [49, 50]. This means that they can identify true-positive TB cases while minimizing false-positive results. The use of deep-learning algorithms has also led to improved efficiency and speed in TB detection [49, 50]. The automated analysis of chest X-ray images allows for processing large datasets in a shorter time, thus enabling faster diagnoses [49, 50]. The quality of deep learning models depends on the frequent training of the datasets [49, 50]. Sufficiently large and diverse datasets that encompass both TB and non-TB cases are crucial for training models that can deliver reliable results [49, 50]. Deep-learning models have shown promising results for detecting extrapulmonary TB [49, 50]. This is important, because TB can also affect bones, kidneys, and the central nervous system [49, 50]. While the use of deep learning for automatic TB detection is promising, it has been emphasized that these models should serve as supportive tools for physicians rather than a replacement for thorough clinical assessment and a definitive diagnosis [49, 50]. These previous studies highlight the potential of enhanced deep-learning models for automatic TB detection in chest X-ray images [21, 45 − 50]. The ongoing development of this technology could contribute to improving the efficiency of TB screening, supporting the fight against TB.

### Xpert MTB/RIF®-assays (Xpert)

The results of previous studies on the Xpert MTB/RIF assay indicate that it is a highly effective diagnostic tool for detecting TB and rifampicin resistance [24, 56, 57]. The assay has showed improved sensitivity and specificity compared to traditional methods [24, 56, 57]. It has also exhibited the ability to detect rifampicin resistance [57]. The Xpert MTB/RIF assay has been widely implemented in many countries and has significantly contributed to the early detection and management of TB cases [57]. The assay is a molecular diagnostic test that utilizes the GeneXpert technology to detect *Mycobacterium tuberculosis*. The assay helps discover rifampicin resistance to *Mycobacterium tuberculosis* [24, 56, 57]. The Xpert MTB/RIF assay has consistently shown excellent sensitivity and specificity, which means that it can accurately identify individuals who have TB infection [24, 56–58]. Furthermore, the Xpert MTB/RIF assay has the added advantage of detecting rifampicin resistance [57]. As a result of its proven efficacy, the Xpert MTB/RIF assay has been widely adopted and implemented in many countries, especially those with a high burden of TB [8]. Its utilization has significantly improved the speed and accuracy of TB diagnosis, enabling early treatment initiation and reducing transmission rates [57]. It is worth noting that, while the Xpert MTB/RIF assay has revolutionized TB diagnostics, ongoing research and development continue to refine and enhance molecular testing methods for TB detection and drug resistance profiling [8].

### Routine passive case-finding and active case-finding using mobile X-ray machines

Several studies have investigated the effectiveness of routine passive and active case-finding using mobile X-ray machines for TB detection [25, 59, 60]. The results of these studies have shown promising outcomes. Routine passive case-finding involves identifying TB cases when individuals seek treatment for TB-related symptoms or other health issues [25, 59, 60]. It relies on the existing healthcare system to diagnose and treat TB. In comparison, active case finding aims to proactively detect TB cases by actively screening high-risk populations such as people living in close proximity to a person with TB or in crowded environments [25, 59, 60]. Studies have demonstrated that routine passive case-finding alone may lead to missing a significant number of TB cases [25, 59, 60]. However, when combined with active case-finding using mobile X-ray machines, the detection rate of TB increases [61, 62]. Mobile X-ray machines enable quick and accessible radiographic examinations, allowing healthcare workers to identify TB-related abnormalities in the lungs and provide timely diagnoses [61, 62]. The use of mobile X-ray machines for active case-finding has led to promising results in terms of improving TB detection rates, especially among prisoners, homeless individuals, and migrants [61, 62]. By bringing diagnostic capabilities to the field and reaching remote areas, mobile X-ray screening can contribute to early TB detection and prompt treatment initiation, reducing transmission and improving patient outcomes [61, 62]. These previous studies highlight the potential of integrating mobile X-ray machines into TB control programs as a part of active case-finding strategies [61, 62]. However, further research and implementation studies are needed to assess the cost-effectiveness, feasibility, and impact of such interventions in relation to TB control at a large scale. Mobile X-ray machines offer several advantages in the context of TB control [61, 62]. They provide a portable and efficient means of conducting chest X-rays, which are crucial for diagnosing pulmonary TB [61, 62]. Using mobile X-ray machines, healthcare workers can reach communities that have limited access to healthcare facilities or areas where transportation barriers exist [61, 62]. This is particularly relevant in rural or remote regions, where conventional radiographic services may be scarce [61, 62]. Active case-finding using mobile X-ray machines targets high-risk populations, including contacts of TB patients, individuals in crowded settings such as prisons and shelters, and marginalized communities [61, 62]. This strategy enables the detection of TB cases at early stages, even in those with minimal or no symptoms, thereby facilitating prompt treatment initiation and reducing transmission rates [61, 62]. By conducting X-rays in these populations, healthcare workers can identify TB cases at an early stage, including those with minimal or no symptoms, enabling prompt treatment initiation and reducing transmission rates [61, 62]. Previous research has indicated that active case-finding using mobile X-ray machines can lead to increased case-detection rates [61, 62]. Similarly, studies in urban slums and refugee camps have demonstrated the effectiveness of active case-finding using mobile X-ray machines for detecting TB cases [63]. However, implementing active case-finding with mobile X-ray machines comes with its own set of challenges [61]. The cost of the equipment, training healthcare workers, and interpreting the X-ray images require careful consideration [61]. Furthermore, the integration of mobile X-ray screening into existing healthcare systems needs to be appropriately planned to ensure sustainability and effective referral systems for further diagnostic confirmation and treatment [61]. Despite these challenges, the potential benefits of integrating mobile X-ray machines into TB control programs are significant [25, 59–63]. Early detection of TB through active case-finding can help prevent the spread of the disease, reduce morbidity and mortality, and contribute to achieving global TB control targets [25, 59–63]. Therefore, the use of mobile X-ray machines for active case-finding has exhibited potential for improving TB detection rates, especially in hard-to-reach populations [25, 59–63]. By bringing diagnostic capabilities to the community, these machines can facilitate timely diagnosis and treatment initiation [25, 59–63]. However, careful planning, resource allocation, and further research are essential to fully understand the impact and feasibility of integrating mobile X-ray machines into routine TB control strategies [25, 59–63].

### Nucleic acid amplification tests

Nucleic acid amplification tests (NAATs) have shown promise for TB screening according past studies [26, 64, 65]. This test identifies *Mycobacterium tuberculosis* genetic material. It offer improved sensitivity and specificity compared to conventional methods [26, 64, 65]. They have the potential to provide rapid and accurate detection of TB, allowing for earlier treatment initiation and reduced transmission rates [26, 64, 65]. However, further research and validation are necessary to establish their clinical utility and widespread implementation for TB diagnosis and control programs [26, 64, 65]. These tests are designed to identify specific regions of the bacterial DNA or RNA and provide highly sensitive and specific results [66]. The polymerase chain reaction (PCR) and loop-mediated isothermal amplification (LAMP) have been developed for tuberculosis screening [67]. Further, NAATs offer several advantages over traditional methods such as microscopy and culture [68]. They have higher sensitivity, which means that they can detect lower concentrations of the TB bacteria even in individuals with paucibacillary (low bacterial load) disease [68]. NAATs also provide faster results, with some tests producing outcomes within a few hours [68]. This rapid turnaround time enables the prompt initiation of TB treatment, reducing the risk of disease progression and transmission [68]. There are still some limitations to consider for NAATs. While NAATs require sophisticated laboratory facilities and trained personnel for accurate and reliable testing, their deployment is increasingly feasible even in resource-limited settings due to advancements in increasingly feasible even in resource-limited settings due to advancements in portable and simplified technologies [69]. They may also involve higher costs compared to conventional methods, which can pose challenges for their implementation in resource-limited settings [70]. Furthermore, while NAATs have demonstrated good performance in research studies [26, 64–70], their real-world effectiveness and impact on TB control programs need further evaluation. Validation studies in different populations and settings are necessary to assess their performance in diverse scenarios. In summary, NAATs have shown promise with respect to improving the diagnosis of TB. Their higher sensitivity, faster results, and potential for early detection can contribute to more effective TB management. Continued research and implementation efforts are crucial for optimizing their use and integrating them into TB control strategies worldwide.

### Development of an immunodiagnostic rapid test for the early detection of TB

The emergence of an immunodiagnostic rapid test for the early detection of TB has made enormous progress after the past investigations [71–73]. The immunodiagnostic rapid test has high sensitivity and specificity for TB diagnosis [71–73]. Moreover, the immunodiagnostic rapid test can analyze sputum and blood, allowing for easy collection and testing in different clinical settings [71–73]. Its quick turnaround time provides rapid results, enabling early detection and timely treatment initiation [71–73]. The development of this immunodiagnostic rapid test addresses the challenges faced in resource-limited settings. The ongoing research and development of this immunodiagnostic rapid test can lead to improving TB diagnosis globally.

### Early detection of pre-extensively drug-resistant TB with line probe assays in countries with a high TB burden

Early detection of pre-extensively drug-resistant TB (pre-XDR TB) using line probe assays has been observed in countries with a high burden of TB [27, 74, 75]. These assays have shown significant potential for identifying drug-resistant strains of TB at an early stage [27, 74, 75]. The use of line probe assays offers the possibility to improve the management and control of TB in these high-burden settings [27]. Several studies have investigated the use of line probe assays as a diagnostic tool for the early detection of pre-XDR TB in regions that were heavily affected by TB [27, 76]. These assays are molecular tests that can rapidly identify specific genetic mutations associated with drug resistance in *Mycobacterium tuberculosis* [27, 76]. The results of these studies have yielded favorable outcomes [27, 76]. Line probe assays have demonstrated the ability to accurately detect drug-resistant strains of TB [27, 76]. This early detection is crucial for promptly initiating appropriate treatment strategies [27, 76]. By identifying drug-resistant strains early on, line probe assays enable healthcare providers to prescribe targeted drug regimens that are more effective against these resistant strains [27, 76]. Moreover, the early detection of pre-XDR TB using line probe assays has the potential to enhance TB control efforts in high-burden countries [27, 76]. Overall, the findings from previous studies highlight the significant impact that line probe assays can have for TB management and control in countries with a high occurrence of the disease [27, 74–76]. By enabling early detection of drug-resistant strains, these assays have the potential to improve patient outcomes, reduce transmission, and contribute to the global efforts to combat TB [27, 74–76].

### Novel, fast (within hours) culture-free diagnostic method with an ultra-sensitive enzyme-linked immunosorbent assay for the detection of live *Mycobacterium tuberculosis* with high sensitivity

The results of a past study showed that the developed enzyme immunoassay (ELISA) diagnostic method had remarkable sensitivity [26]. The highly sensitive ELISA recognized living *Mycobacterium tuberculosis* cells with a sensitivity of 86.9% [26]. Furthermore, the assay showed excellent specificity by accurately distinguishing TB from non-tuberculous bacteria [26]. Cross-reactivity with non-target species was minimal, underscoring the specificity of this test [26].

#### Detection sensitivity

The ultra-sensitive ELISA method demonstrated exceptional sensitivity with regard to detecting live *Mycobacterium tuberculosis* cells [26]. It consistently detected as low as 330 colony-forming units (CFUs) per milliliter of sample [26]. This sensitivity surpassed that of conventional culture-based techniques, which often require higher CFU counts for detection [26]. The ability to detect *Mycobacterium tuberculosis* at such low concentrations is crucial for early diagnosis and intervention [26].

#### Comparison with conventional methods

In comparative studies involving conventional culture-based methods, the ultra-sensitive ELISA exhibited a significantly higher detection rate for live *Mycobacterium tuberculosis* cells [26, 77]. The ELISA-based approach also eliminated the need for lengthy incubation periods, allowing for rapid and timely diagnoses [26, 77].

#### Specificity and cross-reactivity

The assay exhibited minimal cross-reactivity with non-target species, further supporting its specificity [26]. This specificity is crucial for minimizing false-positive results and ensuring accurate diagnoses [26].

#### Clinical sample analysis

The ultra-sensitive ELISA identified TB in sputum, bronchoalveolar lavage fluid, and blood samples [26]. This versatility enhances its potential for widespread implementation in healthcare settings [26].

#### Time efficiency

Compared to conventional culture-based methods, the ultra-sensitive ELISA offered a substantial reduction in turnaround time [26]. The ELISA method provided results within a few hours, enabling the prompt initiation of the appropriate TB treatment and reducing the risk of disease transmission [26].

#### Cost-effectiveness

An economic evaluation of the ultra-sensitive ELISA method revealed considerable cost-effectiveness compared to conventional culture-based techniques [77]. The reduced turnaround time and simplified workflow contribute to potential cost savings, making this method an attractive option for resource-limited settings where TB is prevalent [77].

These results support the novel highly sensitive ELISA-based diagnostic method as an efficient, sensitive, specific and inexpensive tool for early detection of TB [26, 77]. Further validation studies on larger and diverse clinical cohorts are warranted to evaluate the performance and clinical utility of this innovative diagnostic approach.

## Discussion

This systematic review on the early detection of TB distinctively contributes to the existing literature by offering a comprehensive examination of a variety of diagnostic approaches, ranging from traditional methods to advanced technological interventions. Unlike previous reviews that might have focused on singular aspects of TB detection or specific diagnostic tools [6, 12], our study provides an integrated analysis of multiple methods, including the latest advancements such as deep-learning models and molecular assays. Comparatively, our systematic review stands out in its emphasis on the integration of artificial intelligence (AI) in TB diagnostics. This focus is particularly relevant in the context of existing literature, where there has been a growing interest in the application of AI in medical diagnostics [21]. Our study not only highlights the potential of AI in enhancing the accuracy and efficiency of TB diagnosis but also contrasts these advanced methods with traditional techniques, offering a unique perspective on the evolution of TB diagnostic practices. In terms of methodology, our systematic review encompasses a broader range of recent studies, including those published up to June 2023. This approach contrasts with many existing reviews and meta-analyses that may have limitations in terms of the recency of the studies included [43, 53]. Our comprehensive literature search and inclusion of the latest research add a contemporary dimension to our systematic review, making it a valuable addition to the field. Another distinguishing aspect of our systematic review is the incorporation of comprehensive health education as a key component in TB control. This holistic approach aligns with current global health strategies but is often underrepresented in other literature reviews focused solely on diagnostic methods. By highlighting the importance of education and community engagement, our systematic review underscores the need for a multifaceted approach to TB management. When compared to existing meta-analyses [56, 64], our review provides a more narrative and qualitative synthesis of the literature, rather than a quantitative analysis. This approach allows for a broader discussion of the implications of various diagnostic methods and their practical applications in different contexts. Theoretically, our systematic review contributes to a nuanced understanding of the comparative efficacy of different TB detection methods. This aspect is particularly valuable for clinicians and public health policymakers who are looking to make informed decisions about the adoption of new technologies and strategies in TB control. Future research directions suggested by our systematic review, particularly in the effective implementation of AI-based tools in diverse clinical settings and low-resource environments, also set it apart from other reviews. These areas have been less explored in existing literature and present opportunities for significant advancements in TB control.

Several studies have investigated the effectiveness of TB screening programs in relation to identifying cases within the population [18, 28–38]. These studies have illustrated that screening for TB can contribute to early case detection and reduce disease transmission [18, 28–38]. Various screening methods for TB have been analyzed such as X-rays [36], NAATs [26], and immunodiagnostics [71–73].

This systematic review acknowledges the predominant reliance on the study by Wang et al. (2016) [10], a decision driven by the comprehensive depth and methodological rigor it offers on specific aspects of early TB detection. Despite an extensive literature search, this source stood out as the most relevant and exhaustive, thereby becoming a principal reference for this manuscript. It is important to note that such reliance may reflect the current gaps in literature, indicating a nascent stage of research in this area. This limitation is recognized within our systematic review, as we understand that it may influence the breadth of our conclusions. We have attempted to contextualize the findings of the study by Wang et al. (2016) within the broader scope of TB early detection, underscoring the need for further research in this field [10]. The systematic review employs a qualitative synthesis approach, interpreting the findings of the study by Wang et al. (2016) [10] within existing theoretical and conceptual frameworks, thus offering a nuanced understanding while advocating for more diverse research outputs. The frequent citation of the study by Wang et al. (2016) [10] not only highlights its significance as a seminal work in the field but also serves as a clarion call for further comprehensive studies, emphasizing the vast potential for future research endeavors to build upon its foundational findings. Through this systematic review, we aim to contribute to the discourse on TB early detection, while acknowledging the need for a more diversified research landscape in future studies.

One of the pioneering TB early detection studies by Daum et al. (2015) used a combination of symptom screening and molecular testing to identify individuals at risk of TB [78]. The authors found that this integrated approach significantly increased the sensitivity and specificity of TB diagnosis compared to conventional methods [78]. The study has a few important limitations, including a small sample size. It focused on a specific population. The results of the study could affect the generalizability of the data.

A past study examined the usefulness of chest X-rays as a tool for early detection of TB [79]. The study employed a large cohort and utilized advanced imaging techniques to improve the accuracy of TB diagnosis [79]. The findings reflected promising outcomes, with chest X-rays showing high sensitivity with regard to detecting TB-related abnormalities [79]. However, the study did not examine cost-effectiveness [79].

A valuable strategy for TB detection is to identify individuals who have been in close association with TB infected patients. Multiple studies have examined the effectiveness of screening TB contacts [19, 39–42]. It has been demonstrated that this approach can help identify asymptomatic cases and cease disease transmission [19, 39–42].

Educating and training patients and their household contacts play a crucial role in TB control. Studies have shown that comprehensive health education can increase awareness of the disease, improve medication adherence, and reduce the risk of transmission [23, 44, 53].

Utilizing X-ray imaging coupled with in-house deep-learning models for automatic TB detection has the potential to enhance screening efficiency and accuracy [21, 45–50]. This method can facilitate early identification of TB cases and expedite diagnosis [21, 45–50].

The IGRA is a method for diagnosing TB that measures the immune system’s response to certain TB-specific antigens. Studies have demonstrated that the IGRA exhibits high accuracy with respect to detecting latent TB infections and serves as an alternative to other diagnostic tests [22, 51–55].

Routine passive case-finding through raising awareness regarding TB symptoms and active case-finding utilizing mobile X-ray machines have proven to be effective strategies for identifying TB cases in various populations [25, 59–63]. These approaches allow for early detection and prompt treatment initiation [25, 59–63].

NAATs are molecular diagnostic tests. These tests detect the genetic material of *Mycobacterium tuberculosis*. Studies have highlighted the sensitivity and specificity of NAATs for TB diagnosis, especially in cases where traditional culture-based methods may be challenging [26, 64–70].

Research efforts have also focused on developing immunodiagnostic rapid tests for early TB detection [71–73]. These tests aim to provide quick and accurate results, allowing for timely treatment initiation and reducing disease spread [71–73].

Line probe assays represent a diagnostic method that detects specific proteins or substances associated with *Mycobacterium tuberculosis* [27, 74–76]. Its application for the early detection of pre-XDR TB in high TB-burden countries has shown encouraging results.

In addition, researchers have developed a culture-free diagnostic method. They used highly sensitive ELISA to detect live *Mycobacterium tuberculosis* [26]. This innovative approach offers high sensitivity and could improve the detection of TB cases [26]. This innovative approach offers high sensitivity and could improve the detection of TB cases [26].

Another notable study by Perumal et al. (2021) focused on the use of novel biomarkers for the early detection of TB [80]. The researchers identified specific immune-response markers that exhibited the capacity to distinguish active TB cases from latent infections [80]. The study employed rigorous statistical analysis and involved a diverse study population [80]. Although the results have been hopeful, larger, multi-center studies are needed to validate these biomarkers for clinical use [80].

A recent meta-analysis by Vengesai et al. (2021) aimed to summarize the findings of multiple studies on early TB detection methods [81]. The investigation included work using various diagnostic devices such as molecular tests, serological tests and radiological imaging [81]. The meta-analysis revealed significant heterogeneity among the examined studies [81]. Nonetheless, the study emphasized the importance of continued research efforts and standardized protocols for early TB detection.

The reviewed studies underscore the ongoing research efforts associated with the early detection of TB, addressing diverse aspects such as symptom screening, imaging techniques, biomarker identification, and diagnostic tool evaluation [18–27]. While each study contributes valuable insights, it is evident that further research is necessary to refine existing methods and establish robust diagnostic protocols [18–27]. Future studies should aim to consider larger sample sizes, more diverse populations, and comparative analyses to effectively guide clinical practice.

The results of these studies showed the importance of implementing different screening methods, using modern technologies and providing comprehensive training to improve TB detection and control. These cornerstones have the potential to improve early detection of TB, reduce disease transmission and contribute to better TB management worldwide.

## Limitations

Despite the significant advances in early detection of TB, there are limitations in these studies that provide insights for further research and improvement:

1. Generalizability of Findings: Many studies included in the systematic review focus on specific populations or settings. The applicability of the results is unlikely to generalize to broader and diverse populations.
2. Variability in Study Designs: The studies reviewed employ a range of methodologies and designs, which may result in varying levels of evidence strength. This heterogeneity can affect the overall conclusions drawn from the review.
3. Limited Evaluation of Cost-Effectiveness: Few studies address the economic aspects of TB detection methods, such as cost-effectiveness, affordability, and resource allocation, especially in resource-limited settings.
4. Potential for Bias in Literature Search: The systematic review’s reliance on certain databases and English language publications may have excluded relevant studies in other languages or from other databases, leading to potential selection bias.
5. Limited Discussion on Practical Implementation: The systematic review provides limited insight into the practical challenges of implementing these TB detection strategies, such as training requirements, infrastructure needs, and acceptance by healthcare professionals and patients.
6. Insufficient Focus on Long-Term Outcomes: There’s a lack of emphasis on the long-term impact of early TB detection methods, including patient outcomes, disease transmission rates, and overall public health implications.
7. Overemphasis on Technological Solutions: While advanced diagnostic methods are highlighted, there may be an overemphasis on technology-driven solutions, potentially overlooking the importance of basic healthcare services and public health strategies.
8. Lack of Consensus on Diagnostic Criteria: The systematic review reflects a lack of standardized criteria or protocols for TB screening and diagnosis, which could lead to variability in practice and effectiveness.
9. Challenges in Contact Tracing and Education: Despite mentioning the importance of contact tracing and education, the review does not thoroughly address the practical challenges and limitations inherent in these strategies.
10. Dependence on Clinical and Laboratory Infrastructure: Many of the diagnostic methods discussed require substantial clinical and laboratory infrastructure, which may not be feasible in all settings, especially in low-resource environments.
11. Potential for Diagnostic Error: The systematic review does not extensively address the possibilities of false positives or negatives associated with various TB diagnostic methods, which could have significant implications for patients and TB control efforts.
12. Need for Further Research: The systematic review concludes with a call for further research, indicating that current knowledge and technologies for TB detection are not yet fully developed or understood.

## Conclusions

There is a brief summary of the results discussed above:

1. **TB Screening**: Screening individuals for TB helps facilitate early detection and containment of the disease. X-rays, NAATs, and immunodiagnostic approaches have been investigated to enable accurate diagnosis.
2. **Screening TB Contacts**: Screening of people who have been in contact with TB patients is of significant utility in detecting latent TB infection and detecting active TB as early as possible. Screening programs for TB contacts have been shown to be effective.
3. **Comprehensive Health Education for Household Contacts**: Studies have demonstrated that providing comprehensive health education to household contacts of TB patients can improve their awareness of TB. It can also reduce risk behaviors. Comprehensive health education can support community engagement in TB control.
4. **X-Ray for Screen-Active TB with In-House Deep-Learning Model for Automatic TB Detection**: The use of X-rays in conjunction with an in-house deep-learning model for automatic TB detection shows promising results. This method allows for rapid and reliable diagnosis of active TB and can enhance the efficiency of screening programs.
5. **IGRA**: The whole-blood IGRA is a diagnostic test used to identify latent TB infections. This test has high sensitivity and specificity and reliable results.
6. **Routine Passive and Active Case-Finding Using Mobile X-Ray Machines in TB**: The use of mobile X-ray machines for active case-finding in TB has proven effective, particularly in regions with limited access to healthcare. This method enables the early diagnosis and treatment of TB cases.
7. **NAATs**: NAATs can quickly detect *Mycobacterium tuberculosis*. These tests can be helpful in screening of TB.
8. **Ultra-Sensitive ELISA**: The ELISA is a very accurate technique for the detection of TB. By utilizing specific antibodies, this technique can target and detect TB with exceptional sensitivity. The ultrasensitive nature of ELISA enables the detection of even minute quantities of the bacterium, thus contributing to enhanced life safety and quality.

These studies contribute to improving TB screening, detection, and control strategies. Early detection of TB is crucial for effective disease management. There is a need for further research and validation to improve their sensitivity, specificity. Combining multiple diagnostic approaches, such as coupling imaging techniques with molecular tests, may enhance early-detection accuracy. Future efforts should focus on developing affordable, and rapid diagnostic tools for early detection of TB.

## Data Availability

All data are included in the manuscript.
